# Accuracy and reliability of retinal photo grading for diabetic retinopathy: Remote graders from a developing country and standard retinal photo grader in Australia

**DOI:** 10.1371/journal.pone.0179310

**Published:** 2017-06-20

**Authors:** Fakir M. Amirul Islam

**Affiliations:** 1 Department of Statistics, Data Science and Epidemiology, Faculty of Health, Arts and Design, Swinburne University of Technology, Hawthorn, Victoria, Australia; 2 Organisation for Rural Community Development (ORCD), Dariapur, Narail, Bangladesh; Soochow University Medical College, CHINA

## Abstract

**Background:**

To evaluate the accuracy and reliability of fundus retinal photos graded by local graders in Bangladesh with those graded by an expert at the Centre for Eye Research Australia (CERA) in the context of mass scale diabetic retinopathy (DR) screening in Bangladesh.

**Methods:**

A population-based cross-sectional study of 3,104 adults identified 213 (7.2%) eligible patients with diabetes of age ≥ 40 years in 2012–2013. Retinal photographs were collected using a non-mydriatic digital fundus retinal camera and a two-field imaging protocol. The photos were graded by two remote graders (G1 and G2) who were trained by a retinal specialist (RS) in Bangladesh, by the RS himself, and by a Centre for Eye Research Australia (CERA) grader. The local graders up skilled their grading ability by comparing 30% of the photos graded by the CERA grader with their own grades. Learning from that exercise was applied to the remaining 70% of photos, which were re-graded. Reliability and accuracy of grading amongst the graders were reported using cross tabulation, inter- and intra-grader reliability, and with sensitivity and specificity.

**Results:**

Of 122 eyes from 61 patients, the mild (R1) DR was estimated to be 14 to 25%, pre-proliferative (R2) DR 4–8%, and proliferative (R3) DR 0.8 to 1.6%, whereas 25%, 8%, 18%, and 15% were found to be ungradable by CERA, RS, G1, and G2, respectively. Of 8 (6.6%) eyes identified as R2 by the CERA grader, 5 (63%), 3 (38%) and 3 (38%) were correctly classified as R2, whereas the rest were classified either as R1 or R3 but none were classified as no DR (R0) or ungradable by the RS, G1 and G2, respectively. After getting experience reviewing the 30% test set graded by the CERA grader, the local graders graded moderate and severe DR with 100% accuracy. After excluding ungradable photos, the sensitivity (specificity) relative to the CERA grader was 82% (88%) before and 80% (93%) after training for G1 and 56% (87%) before and 77% (90%) after training for G2. In case of maculopathy, the CERA grader reported 11.2% eyes with maculopathy, which included 100% of the 4.9% by RS, 6.6% by G1, and 7.4% by G2.

**Conclusions:**

Local graders in Bangladesh are able to grade retinal photos with high accuracy if the DR is at least of a moderate level. With appropriate training and experience, local graders have the ability to contribute significantly to the grading of millions of retinal photos, which required grading in resource- poor countries.

## Introduction

Diabetic retinopathy (DR) is a major complication of diabetes mellitus (DM) which, if left untreated, can result in blindness. Not only is it a cause of blindness, it is also associated with a 2–3 times higher risk of stroke, coronary heart disease, and heart failure independent of cardiovascular risk factors [1, 2]. The amount of DR is increasing as the number of cases of DM increases, especially in Asia, including Bangladesh and sub-Saharan Africa [3, 4]. A recent meta-analysis showed that the prevalence of DM among adults in Bangladesh has increased from 4% in 1995–2000 to 9% in 2006–2010, equating to approximately 13 million people living with diabetes amongst the 160 million people[5]. Amongst the approximately 347 million people worldwide reported to have DM, 34% have some form of DR and about 10% have vision-threatening DR [6, 7]. DR accounts for 4.8% of the 37 million cases of blindness worldwide [8].

Fortunately, DR is preventable with early diagnosis, timely referral for ophthalmic care, and early treatment. However, one of the challenges of treating DR is that it is non-symptomatic in the early stages and does not manifest symptoms until visual impairment occurs [8]. Therefore, periodic screening for early detection of DR, which is universally accepted, needs to be a priority. Currently, however, screening is not uniformly available in developed countries and widespread systematic retinal examination of people with DM is virtually non-existent in Bangladesh and other developing countries [9].

According to the American Diabetes Association (ADA) guidelines, every patients diagnosed with DM needs to have an immediate retinal examination and a follow-up examination every 2 years so long as they are without DR. For those with moderate to severe DR, a follow-up retinal examination should be completed every 3 months after detection. This indicates that on an average, every patient with diabetes need to have a retinal examination once a year. In total, this means that almost 13 million people with diabetes need to be photographed for detection of DR in Bangladesh alone [10]. The process of screening for DR is complex and attention to detail is required if screening efforts are to succeed. Friedman and colleagues [9] conducted a comprehensive study to identify barriers to screening for DR in resource-poor settings and reported that an efficient and accurate system must be in place if there is any hope of screening in this setting. The authors also expressed a number of concerns about such initiatives, including the use of general medical doctors without specialized education and training. [9]. However, the fact is that the human resources for eye care services and number of skilled graders in low resource countries are extremely inadequate, [11, 12] when compared to the targeted ratio of ophthalmologists to population of 1:100,000, as set in 2010 by the World Health Organization (WHO) [13].

Bearing in mind a number of limitations in collecting and grading retinal photos in a resource-poor setting, what can be done to screen large numbers of patients with DM in rural areas where no specialist physicians or retinal specialists are available even at the district level. The optimal solution is for ophthalmic assistants and technicians at the district level to be trained in collecting and grading retinal photos and to establish a referral pathway between technicians and the ophthalmologists in the major cities. However, it is currently unknown whether local technicians are able to collect and grade retinal photos with an acceptable level of accuracy.

The current study aims to check the reliability and accuracy of grading retinal photos by rural technicians in comparison with a grader from the CERA (gold standard) grader. The study also aims to determine if accessing feedback on photos graded by a CERA grader increases the ability of local graders to grade photos accurately.

## Materials and methods

### Study sample

In a cross-sectional study of 3,104 adults aged ≥30 years that aimed to study the prevalence of and risk factors for diabetes [14–17], we identified 220 participants with diabetes. Of those, 213 were 40 years of age or older and eligible to participate in this study. All eligible participants were contacted and asked to attend the Organisation for Rural Community Development (ORCD) centre to collect retinal photographs. Participants were recruited from March to July 2015. Retinal photographs are being obtained using a non-mydriatic digital fundus retinal camera and a two-field imaging protocol. To date, retinal photos from 84 participants have been obtained, and the study is ongoing. For the current study, we have analyzed 244 photos from 122 eyes of 61 participants. The study participants, their characteristics, and recruitment strategy have been described elsewhere [14, 16].

### Retinal photography and grading photos

Photographs were taken using a 45-degree, 6.3 megapixel digital non-mydriatic camera (Canon, Lake Success, NY). Participants were seated in a darkened room. Both eyes of each participant were photographed using a two-photographic fields protocol, the first centered on the optic disc (Field 1) and the second centered on the fovea (Field 2). Standard software was used for image acquisition and archiving (Digital Healthcare Inc. Eye QSL, England). Images were then graded for retinopathy and other retinal diseases. The Early Treatment Diabetic Retinopathy Study (ETDRS) [18], which is considered to be more appropriate to grade for retinal photos as it captures seven fields of the retina, was not used for this intended mass scale screening for DR. The ETDRS grading scheme is very complicated and involves seven pictures of each eye making it impractical for a large-scale screening program in a rural setting. Therefore, a simplified version of ETDRS was used for the current grading purpose [19].

The photos were graded by two trained, local graders-a health technologist (G1) and a bachelor of arts graduate (G2)-and a retinal specialist who provided training to the local graders. Photos were subsequently graded by a senior grader at CERA in Melbourne, Australia, as per same DR grading protocol [19].

### Training for the local graders

The local graders received 2 months training from a retinal specialist at the Dhaka Vision Eye Hospital. They received training on collecting and grading retinal photos, basic anatomy of the eyes, and basic understanding of eye care. They also received training from a CERA grader, which is considered to be the gold standard. The CERA grader graded photos with a comprehensive explanation for each of the photos, and those were sent to the local graders to improve their grading skills. The local graders divided the photos into two groups: 1) a small group of 64 photos from 32 eyes (i.e., test set to increase grading skills) and 2) a large group of 180 photos from 90 eyes (i.e., experimental set to compare grading accuracy). The local graders reviewed grading explanations provided by the CERA grader and compared their graded photos with the test set to improve their grading skills. By doing so, the local graders graded the experimental set again. Grading for second time took place 3 months after the first grading, which negates the possibility of past experience influencing grading ability. In total, photos were graded by the two local graders, one retinal specialist (RS) and by a CERA grader who has more than 15 years of experience in DR and retinal vessel calibre grading.

### Diabetic retinopathy

Retinopathy was classified according to a modified version of ETDRS by Shotliff and Duncan [19]: Summary of grading and management criteria. DR level was defined as:

Level R0 = None or no DR

R1 = Microaneurysms (MA), retinal hemorrhages (H) and any exudates

R2 = Intra-retinal microvascular abnormalities (IRMA), venous beading (VB), venous loop or reduplication, multiple deep, round or blot hemorrhages, and cotton wool spots (CWS)

R3 = New vessels on disc (NVD), new vessels elsewhere (NVE), pre-retinal or vitreous hemorrhage, pre-retinal fibrosis ± tractional retinal detachment

### Maculopathy

M0 = no maculopathy

M1 = Maculopathy present; exudate within 1 disc diameter (DD) of the centre of the fovea, or circinate or group of exudates within the macula, or retinal thickening within 1DD of the center of the fovea (if stereo available) or any microaneurysm or hemorrhage within 1DD of the centre of the fovea (only if associated with a best visual acuity of ≤6/12)

### Ethics approval

This research adhered to the tenets of the Declaration of Helsinki and the research protocol was approved by Human Research Ethics Committee at the Swinburne University of Technology (RES 2015/66). We obtained written consent from participants who were able to sign their name, and a finger print was obtained from those who were unable to do so (47%). In the case of finger print consent, the data collector provided a counter signature for the participants. Participants were informed of their right to withdraw from the study at any stage or to request their data be excluded from analysis.

### Statistical analysis

The prevalence of DR estimated by different graders was reported using simple descriptive statistics and presented graphically. Cross tabulation was used to report agreement of level of DR and maculopathy between CERA and other graders. Intra- and inter-grader reliability were reported using correlation coefficients. Accuracy was assessed using sensitivity and specificity, calculated using the formula for sensitivity = (a/(a+c)) and specificity = (d/(b+d)). In the formula “a” equals the number of DR or maculopathy graded correctly by both graders; “b” equals the number of DR or maculopathy correctly classified by the CERA grader but misclassified by the other grader; “c” equals the number of non-DR or no maculopathy correctly classified by the CERA grader but misclassified by other grader; and “d” equals the number of non-DR or no maculopathy correctly classified as non-DR by both the CERA and local graders. The primary objective was to report the percentage of correctly classified DR or maculopathy by the local graders compared to those graded by the CERA grader. The statistical software SPSS (SPSS Inc., version 21) was used for data analyses.

## Results

The ratio of male and female participants who were included in the study (21 (35%) male and 40 (65%) female) was similar to that who were not included in the study (55 (36%) male and 97 (64%) female) from the eligible 213 participants. The mean age of the included participants was 52 years (standard deviation = 8, range = 42–71), which was also similar to the participants who were not included in the study 54 (11, 40–78) (Table 1).

**Table 1:** Age and sex of patients with diabetes included and those who were not included in the diabetic retinopathy study.

	^a^Patients not included, n = 152	^b^Patients were included, n = 61
Sex		
Female, n (%)	97 (64)	40 (65)
Male, n (%)	55 (36)	21 (35)
Age in years		
Mean (SD, min.-max.)	54 (11, 40–78)	52 (8, 41–72)

^a^at least 20% of DM patients were not interested to participate the DR study.

^b^The study is ongoing for prevalence of and risk factors for DR study.

**Fig 1:**
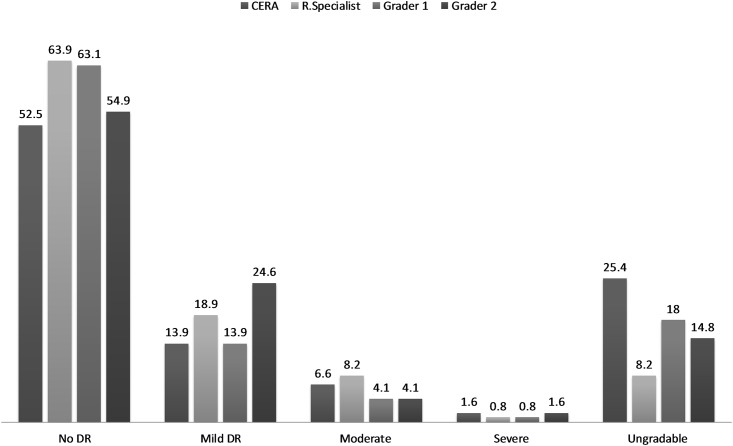
DR (%) graded by CERA grader, retinal specialist (R. Specialist) and the local graders (Grader 1 and Grader 1).

Of 244 photos, the percentage of DR lesions (R1) (i.e., micro-aneurysm or retinal hemorrhage or exudates) was estimated to be 14%, 19%, 14% and 25% of eyes; preproliferative DR (R2) was 7%, 8%, 4% and 4%; and proliferative DR (R3) was 1.6%, 0.8%, 0.8% and 1.6% eyes which were graded by the CERA, RS, and local graders G1 and G2, respectively. The ungradable photos were 25%, 8%, 18% and 15% by the CERA, RS, and local graders G1 and G2, respectively (Fig 1).

Of 64 (52.5%) eyes identified with no retinopathy (R0) by the CERA grader, the correctly classified eyes were 83%, 88% and 72% by RS, G1 and G2, respectively. Of 17 (13.9%) identified as R1 of DR by the CERA grader, 5 (29%), 7 (41%) and 8 (47%) were correctly classified as R1, whereas 3 (18%), 2 (12%) and 2 (12%) were classified as R2 by RS and G1 and G2, respectively. Of 8 (6.6%) identified as R2 by the CERA grader, 5 (63%), 3 (38%) and 3 (38%) were correctly classified as R2, whereas the rest were classified either as R1 or R3 and none were misclassified as R0 or ungradable. After receiving training through a test set of 64 eyes, photos identified as R2 and R3 by the CERA grader were perfectly classified by the local graders (Tables 2 and 3). Of 78 (63.9%) eyes identified with no retinopathy by the RS, the correctly classified eyes were 86% and 78% by the G1 and G2, respectively. Of 23 (18.9%) identified as R1 category by the RS, 6 (26%), 13 (57%) were correctly classified as R1 by G1 and G2, respectively. In cases of R2 and R3, more than 90% were classified correctly (Table 4).

**Table 2:** Comparison of diabetic retinopathy graded by the CERA grader, retinal specialist and the local graders before training through the test set.

CERA grader															
	R0 = 64 (52.5%)			R1 = 17 (13.9)			R2 = 8 (6.6)			R3 = 2 (1.6)			U = 31 (25.4%)		
	RS	G1	G2	RS	G1	G2	RS	G1	G2	RS	G1	G2	RS	G1	G2
	R0	R0	R0	R1	R1	R1	R2	R2	R2	R3	R3	R3	U	U	U
R0	53 (82.8)	56 (87.5)	46 (71.9)	9 (52.9)	8 (47.1)	7 (41.2)							16 (51.6)	13 (41.9)	14 (45.2)
R1	11 (17.2)	4 (6.3)	15 (23.4)	5 (29.4)	7 (41.2)	8 (47.1)	2 (25.0)	4 (50.0)	3 (37.5)		1 (50)	1 (50)	5 (16.1)	1 (3.2)	3 (9.7)
R2				3 (17.6)	2 (11.8)	2 (11.8)	5 (62.5)	3 (37.5)	3 (37.5)	1 (50)			1 (3.2)		
R3							1 (12.5)	1 (12.5)	2 (25.0)						
U		4 (6.3)	3 (4.7)							1 (50)	1 (50)	1 (50)	9 (29.0)	17 (54.8)	14 (45.2)

**Table 3:** Comparison of diabetic retinopathy graded by the CERA grader and the local graders after learning from a test set of 32 eyes graded by the CERA grader.

CERA Grader										
	R0 = 47 (52.2%)		R1 = 10 (11.1)		R2 = 4 (4.4%)		R3 = 1 (1.1%)		U = 28 (31.1%)	
	G1	G2	G1	G2	G1	G2	G1	G2	G1	G2
	R0	R0	R1	R1	R2	R2	R3	R3	U	U
R0	42 (89.4)	42 (89.4)	4 (40.0)	6 (60)					14 (50)	11 (39.3)
R1	4 (8.5)	4 (8.5)	5 (50.0)	3 (30)						1(3.6)
R2			1 (10.0)	1 (10)	4 (100)	4 (100)				
R3							1(100)	1 (100)		
U	1 (2.1)	1 (2.1)							14 (50)	16 (57.1)

**Table 4:** Comparison of diabetic retinopathy graded by the retinal specialist and the local graders.

Retinal specialist										
DR	R0 = 78 (63.9%)		R1 = 23 (18.9)		R2 = 10 (8.2)		R3 = 1 (0.8)		U = 10 (8.2%)	
	G1	G2	G1	G2	G1	G2	G1	G2	G1	G2
	R0	R0	R1	R1	R2	R2	R3	R3	U	U
R0	67 (85.9)	61 (78.2)	10 (43.5)	6 (26.1)						
R1	5 (6.4)	12 (15.4)	6 (26.1)	13 (56.5)	5 (50)	5 (50)	1 (100)			
R2			1 (4.3)	1 (4.3)	4 (40)	4 (40)				
R3			1 (4.3)	1 (4.3)				1 (100)		
U	6 (7.7)	5 (6.4)	5 (21.7)	2 (8.7)	1 (10)	1 (10)			10(100)	10 (100)

Of 93 (76.4%) eyes identified without maculopathy by the CERA grader, the correctly classified eyes were 99%, 93% and 94% by the RS, G1 and G2, respectively. Of 14 (11.5%) identified as M1 category by the CERA grader, 6 (43%), 8 (57%) and 9 (64%) were correctly classified by the RS, G1 and G2, respectively. After training was received through the training set of 7 (7.8%) graded as M1 by the CERA grader, 6 (86%) and 7 (100) were correctly classified by G1 and G2, respectively (Tables 5 and 6).

**Table 5:** Comparison of maculopathy graded by the CERA grader, retinal specialist and the local graders before training through the test set.

CERA grader									
	M0 = 93 (76.4%)			M1 = 14 (11.5)			U = 15 (12.3)		
	RS	G1	G2	RS	G1	G2	RS	G1	G2
	M0	M0	M0	M1	M1	M1	U	U	U
M0	92 (98.9)	86 (92.5)	87 (93.5)	8 (57.1)	5 (35.7)	4 (28.6)	6 (40)	1 (6.7)	4 (26.7)
M1				6 (42.9)	8(57.1)	9 (64.3)			
U	1 (1.1)	7 (7.5)	6 (6.5)		1 (7.1)	1 (7.1)	9 (60)	14 (93.3)	11 (73.3)

**Table 6:** Comparison of maculopathy graded by the CERA grader and local graders after learning from a test set of 32 eyes graded by the CERA grader.

CERA	M0 = 71 (78.9%)		M1 = 7 (7.8%)		U = 12(13.3)	
	G1	G2	G1	G2	G1	G2
M0	67 (94.4)	66 (93.0)			2 (16.7)	3 (25)
M1			6(85.7)	7 (100)		
U	4 (5.6)	5 (7.0)	1 (14.3)		10 (83.3)	9 (75)

Intra-class correlation coefficients between the CERA grader and the RS (0.62), G1 (0.70), and G2 (0.63) were good, but these were stronger between RS and the local graders, G1 (0.78) and G2 (0.83), respectively. The intra-class correlation between the local graders G1 and G2 was very strong (0.94). For maculopathy, the intra-class correlation was between 0.78 to 0.84 among the CERA grader, the RS and the local graders (Tables 7 and 8).

**Table 7:** Intra-class correlation coefficient (95% CI) among CERA grader, retinal specialist and local graders in grading DR and maculopathy before training through the test set.

	CERA grader	Retinal Specialist	Grader 1
Diabetic Retinopathy			
Retinal Specialist	0.62 (0.46, 0.73)**		
Grader 1	0.70 (0.58, 0.79)**	0.78 (0.68, 0.85)**	
Grader 2	0.63 (0.47, 0.74)**	0.83 (0.75, 0.88)**	0.94 (0.92, 0.96) **
Maculopathy			
Retinal Specialist	0.80 (0.71, 0.86)**		
Grader 1	0.84 (0.76, 0.88)**	0.76 (0.66, 0.83)**	
Grader 2	0.78 (0.68, 0.84)**	0.83 (0.76, 0.88)**	0.94 (0.91, 0.96) **

** for P<0.001

**Table 8:** Intra-class correlation coefficient (95% CI) among CERA grader and local graders in grading DR after learning from a test set of 32 eyes graded by the CERA grader.

	CERA grader	Grader 1
Grader 1	0.71 (0.56, 0.81)**	
Grader 2	0.77 (0.65, 0.85)**	0.90 (0.80, 0.92)**

** for P<0.001

In grading DR, sensitivity (specificity) between the CERA grader and G1 were 82% (88%) before training and 80% (93%) after training. Sensitivity (specificity) between the CERA grader and G2 were 56% (87%) before training and 77% (90%) after training. In case of maculopathy, the sensitivities were 100% and specificities were above 85% among the CERA grader, the RS, and local graders (Tables 9 and 10).

**Table 9:** Correctly classified retinal photos with and without DR, and with and without maculopathy by CERA grader, retinal specialist and the local graders before training through the test set.

Diabetic Retinopathy						
	Retinal Specialist		Grader 1		Grader 2	
CERA grader	DR +	DR-	DR +	DR-	DR +	DR-
DR+	17	9	18	8	19	7
DR-	11	53	4	56	15	46
Sensitivity	61%		82%		56%	
Specificity		86%		88%		87%
Maculopathy						
CERA grader	Mac.+	Mac.-	Mac.+	Mac.-	Mac.+	Mac.-
Maculopathy+	6	8	8	5	9	4
Maculopathy -	0	92	0	86	0	87
Sensitivity	100%		100%		100%	
Specificity		92%		95%		96%

**Table 10:** Correctly classified retinal photos with and without DR by CERA grader and the local graders in grading DR after learning from a test set of 32 eyes graded by the CERA grader.

CERA grader	Grader 1		Grader 2	
	DR+	DR-	DR+	DR-
DR+	12	3	10	5
DR-	3	43	3	43
Sensitivity	80%		77%	
Specificity		93%		90%

## Discussion

In this first study of reliability and accuracy of grading retinal photos by local graders in a rural district in Bangladesh compared with a senior grader at CERA, we found that local graders are able to grade photos with at least 80% accuracy. The accuracy becomes at least 80% if DR is at the moderate to severe stage by taking trainings from the specialist graders from overseas and by comparing their own grading with the photos graded by the specialist graders. Initially, the interclass correlation of grading photos between the RS and the local graders was about 80%, though it was only about 60% between the CERA and local graders. However, after the local graders were trained using a test set graded by the CERA grader, the interclass correlation increased to more than 70%. In case of maculopathy, interclass correlation coefficients were more than 85% and sensitivity increased more than 20% (56% before and 77% after training). These findings are important given the need to collect and grade retinal photos, per ADA criteria [10], from a large population with DM in Bangladesh and other resource-poor countries.

This research demonstrated that task sharing amongst ophthalmologists and mid-level eye and health care workers in screening and detection of DR is needed, possible, and has the potential to address the current shortage in the eye care workforce. The findings from this research included good reliability and accuracy in grading retinal photos by local graders compared with the CERA grader. Expanding the roles of mid-level health workers and ophthalmologists in major eye hospitals can creating bridges between people with diabetes in the community and help extend eye care to the community. This entails empowering mid-level eye and health care workers and providing them with adequate training, technology, and policy to share specific tasks with ophthalmologists. Task sharing in eye care for people with diabetes could provide benefits of increased access to eye care services and optimum utilization of the health workforce, which are important elements in eye care delivery [11, 12].

The local graders were able to grade photos in the R2 and R3 categories above 90% correctly after gaining skills from the test set graded by the CERA grader. However, there was a reasonable proportion of misclassification in the case of R1 category. The local graders, including the RS, graded the majority of the photos as R0, however, those were graded as R1 by the CERA grader. In fact, tracking small retinal features, such as micro-aneurysms, requires more experience and skill, high recordkeeping accuracy, and competence in grading [20]. Therefore, misclassification in the R1 category by the local graders was anticipated and their accuracy can be expected to increase in reliability as their years of experience increases. Since the R1 category requires the participant to undergo a second retinal examination after a year and R2 and R3 categories need immediate referral to an ophthalmologist, misclassification of a proportion of the R1 category may a less serious issue at the screening stage.

The prevalence of DR in our study was (22.1%), as graded by the CERA grader, and 18.8% to 30.3%, as graded by the RS and local graders. The results from the local graders are comparable with another study conducted in a small sample in Bangladesh (21.6%) [21], the Hoorn study (23.5%) in Netherlands [22], but higher than the Finnish study in Finland (14.0%) and the Chennai Urban Rural Epidemiology Study (CURES) in India (17.6%)[23]. The prevalence of DR graded by the CERA grader would be higher than 22.1% if the grader had been able to grade 100% of the photos. The prevalence of 22.1% was found from 75% of the gradable photos, indicating about 30% would have DR if all photos had been gradable. The higher prevalence of DR in our study compared to the previous study in Bangladesh can be attributed with the fact that our study was conducted in people of age 40 years or older, compared to 25 years and older in another study [21], as DR is consistently associated with older age [21, 22, 24].

Amongst the strengths of our study is the obtainment of the first data investigating the accuracy and reliability of grading retinal photos by local graders in a rural district in Bangladesh, compared with a world class grader at CERA and with a RS in Bangladesh. Non-mydriatic fundus retinal camera, which is considered the gold standard for collecting retinal photos, was used for this study. The potential limitation of our study was use of a two photographic fields protocol, compared to most studies which use seven fields protocol to capture DR lesions [2, 18, 24–26]. The sample size was small and thus the study had insufficient power, caution is necessary before recommending mass scale grading by local graders.

## Conclusions

In conclusion, this study demonstrated that local graders can be trained by retinal specialists in Bangladesh and that grading accuracy increases significantly if local graders are able to access photos with an explanation of grading provided by an expert grader located overseas. This is beneficial because it showed that learning can occur without the physical attendance of rural graders in developed countries, which would involve a huge amount of cost for remote graders. This study also demonstrated that grading by local graders would help address the challenge of the massive increase in diabetes and its complications in rural Bangladesh.

## Supporting information

S1 Dataset Diabetic retinopathy data. (SAV)
